# Hematological Parameters in Sheep: Variability, Determinants, and Applications in Flock Health Management

**DOI:** 10.3390/ani16091295

**Published:** 2026-04-22

**Authors:** Vera Korelidou, Panagiotis Simitzis, Theofilos Massouras, Athanasios I. Gelasakis

**Affiliations:** 1 Laboratory of Anatomy and Physiology of Farm Animals, Department of Animal Science, School of Animal Biosciences, Agricultural University of Athens (AUA), Iera Odos 75 Str., 11855 Athens, Greece; vkorelidou@aua.gr; 2 Laboratory of Animal Breeding and Husbandry, Department of Animal Science, School of Animal Biosciences, Agricultural University of Athens (AUA), Iera Odos 75 Str., 11855 Athens, Greece; pansimitzis@aua.gr; 3 Laboratory of Dairy Science and Technology, Department of Food Science and Human Nutrition, Agricultural University of Athens (AUA), Iera Odos 75 Str., 11855 Athens, Greece; theomas@aua.gr

**Keywords:** hematology, sheep, blood analysis, diagnosis, health monitoring, livestock management

## Abstract

Blood is a key component of organisms, serving numerous functions, including metabolism, innate and humoral responses, and hemostasis. Variations in hematological parameters can indicate the presence of infectious and non-infectious diseases, chronic stress, and other pathological or physiological conditions. Complete blood count testing is common in human and veterinary medicine and, when combined with clinical examination, contributes to disease diagnosis and prognosis and the monitoring of therapeutic progression. Nevertheless, hematological analysis is not routinely performed in sheep due to the lack of case-specific reference intervals, complicating the interpretation of the results. Indeed, hematological parameters may be affected by various non-pathological (environmental, genetic, physiological) and pathological factors, and they require further understanding and relevant adjustments to be universally applicable. Therefore, the objective of this paper is to summarize the existing literature and describe how various pathological and non-pathological factors affect hematological parameters in sheep, thereby supporting their incorporation into health management practices.

## Simple Summary

Hematological parameters combined with clinical examination can be used for disease diagnosis, prognosis, and health monitoring. However, the absence of case-specific standardized reference intervals and the effects of various factors on hematological parameters complicate their interpretation and widespread adoption in sheep health management. The aim of this review paper is to consolidate the existing literature on hematological parameters in sheep and examine pathological and non-pathological factors affecting these parameters.

## 1. Introduction

Sheep farming is an important component of the livestock sector, contributing substantially to the rural economy and providing essential products such as meat, wool, milk, and skin [1]. Over the last 50 years, the global sheep population has grown by only 3.0%, yet the production of sheep milk, meat, and skin has more than doubled [2], reflecting the influence of genetic improvements, production intensification [3], and modernization in animal breeding practices [4]. Nevertheless, the increasing demand for high-quality sheep products has been linked to emerging animal health and welfare issues. At the same time, the deterioration of the epidemiological statuses of existing diseases, as well as the transmission and re-emergence of contagious diseases, underscores the need for robust and efficient disease prevention, surveillance, and monitoring strategies [5].

In this context, diagnostic and screening tests are recognized as essential tools in veterinary medicine, supporting individual and flock health management and disease control [6]. In particular, they facilitate the early diagnosis, risk assessment, and modeling of infectious diseases, as well as epidemiological studies and the mitigation of disease outbreaks [7]. In addition, they play a crucial role in preventing transboundary disease spread, therefore ensuring the safe international trade of animals and products in accordance with international standards [8].

Blood tests are among the most widely used tools in human and veterinary medicine, providing valuable insights into patients’ and animals’ health statuses and potential underlying causative factors [9]. Among blood tests, a complete blood count is the most requested one, typically performed on fully automated analyzers and serving as a first-line screening tool [10]. There are two main categories of automated hematology analyzers. The first includes automated impedance counters, which determine cell counts by measuring changes in electrical impedance as cells pass through detection electrodes. The second one comprises automated flow cytometers, which are the most widely used; these instruments identify and classify cells by detecting the amounts of light absorbed and scattered by them when passing through a laser beam [11]. Hematological parameters can also be determined through manual cellular analysis, which remains the least expensive method for cell counting. However, it is labor-intensive and requires considerable technical expertise. Despite advances in automated technologies, the packed cell volume (microhematocrit) method continues to be used, as it serves as a reference method for validating the performance of hematology analyzers [12].

Hematological parameters can be used for detecting diseases, determining their severity, obtaining prognoses, and monitoring the progression of therapies or diseases affecting the hemopoietic and other systems [13,14]. Many hematological biomarkers used in veterinary practice have been adapted from human medicine [15], with their interpretation relying on reference intervals established from healthy animals [16]. Hematological analysis is routinely performed in small domestic animal diagnostics (e.g., dogs, cats) [17,18]; however, its application in farm animals, and particularly sheep, remains limited due to the cost, the lack of reliable reference intervals across various management systems, and low specificity [19]. Hematological parameters are also affected by a wide range of non-pathological factors (e.g., breed, age, and stage of lactation) [20], which further undermine their diagnostic value. Establishing reference intervals partitioned by breed, age, and stage of production may be expensive and laborious due to the need to collect more data, yet it is imperative in standardizing result interpretation.

Understanding the factors that affect hematological parameters in sheep and establishing reference intervals accordingly could facilitate the early detection of subclinical disease and improve the monitoring and management of health and welfare at both the flock and animal levels, prioritizing further diagnostic investigations. Currently, several studies have demonstrated the effects of animal-related factors, applied management practices, and pathological conditions on hematological parameters [21,22,23,24]; however, the results are sometimes contradictory due to differences in study designs, including variable sheep populations across different geographical locations and management systems. Therefore, the objective of this review paper is to summarize the existing literature on both pathological (e.g., infectious and parasitic diseases, inflammatory processes, metabolic disorders) and non-pathological factors (e.g., environmental, genetic, and physiological) that affect hematological parameters in sheep, aiming to support their effective application in sheep flock health management practices.

## 2. Blood Composition and Hematological Parameters

Blood consists of cellular elements, namely erythrocytes, leukocytes, and thrombocytes, which are commonly referred to as red blood cells (RBCs), white blood cells (WBCs), and platelets, respectively, particularly in clinical and diagnostic contexts [25]. These cells are suspended in plasma, a fluid composed predominantly of water with less than 10% solids, including plasma proteins (albumin, globulins, fibrinogen), coagulation factors, enzymes, hormones, electrolytes, vitamins, and microelements [26]. In ruminants, the total blood volume accounts for approximately 6–7% of the body weight, with RBCs being the most abundant cellular constituents, accounting for one-quarter to one-half of the total blood volume depending on the species, followed by platelets and, lastly, WBCs [14]. The blood color is typically red, imparted by the iron present in hemoglobin, yet variations depend on the degree of saturation of hemoglobin with oxygen [27]. Dark-brown discoloration has been associated with methemoglobinemia, which may arise from exposure to nitrates and nitrites, oak toxicosis, and copper poisoning [28].

### 2.1. Erythrocytes

Erythrocyte parameters include the red blood cell count (RBC), hematocrit (HCT), hemoglobin (HGB), or packed cell volume (PCV), depending on whether values are obtained using hematological analyzers or centrifugation, as well as the mean corpuscular volume (MCV), mean corpuscular hemoglobin (MCH), mean corpuscular hemoglobin concentration (MCHC), and red cell distribution width (RDW) [27] (Table 1). Erythrocytes are produced in the bone marrow, and their fundamental functions are to support oxidative metabolism by delivering oxygen to the tissues [29], transporting carbon dioxide to the lungs, and buffering hydrogen ions [30]. In sheep, erythrocytes are biconcave, discoid-shaped, anucleate cells that stain reddish to orange, similarly to those of other mammals. Their average diameter is approximately 4.5 μm, which is smaller than that in cows (5.5 μm) and larger than that in goats (3.2 μm) [31]. Notably, sheep and goats, along with camelids, possess some of the smallest erythrocytes among domestic species, possibly indicating an adaptive feature associated with breeding in high-altitude environments, where oxygen availability is reduced [27,32]. Thus, species-specific thresholds should be used in automated hematology analyzers to avoid the misclassification of small erythrocytes as thrombocytes [33].

### 2.2. Leukocytes

Leukocytes constitute a small fraction of the total blood volume [34] and represent an integral part of the immune system. They participate in both innate and adaptive immune responses by mediating inflammatory, cellular, and humoral reactions against pathogens and injuries [35]. Leukocytes are broadly classified into two main categories based on the presence or absence of microscopic granules in their cytoplasm: (i) granulocytes (polymorphonuclear cells), which include neutrophils, eosinophils, and basophils, and (ii) mononuclear cells, comprising lymphocytes and monocytes [36]. With the exception of lymphocytes, all leukocyte types are produced and undergo maturation in the bone marrow [33].

Granulocytes are differentiated by their size, the nucleus morphology, and the staining characteristics of their cytoplasmic granules when evaluated using Wright- or Giemsa-based stains [35,37]. Mature segmented neutrophils in sheep resemble those of goats, measuring 10–15 μm in diameter and possessing a large, multilobulated nucleus. Eosinophils are slightly larger than neutrophils and basophils and contain round, refractile eosinophil granules that stain purple to red, depending on the staining method. Finally, basophils are smaller (8–10 μm diameter), with a round shape, a less segmented nucleus than neutrophils, and a pale blue cytoplasm that contains numerous basophilic granules that may obscure the nucleus [38].

Among mononuclear cells, lymphocytes are readily distinguished from monocytes; they are small to intermediate in size (6–9 μm), with a round to oval nucleus, clumped chromatin, and scant pale to moderately blue cytoplasm. Contrarily, monocytes tend to be larger, with a round-to-oval shape and pale blue–gray cytoplasm, and may contain cytoplasmic vacuoles and fine azurophilic granules [38].

Adult sheep have the lowest mean total white blood cell count (WBC) among domestic species (approximately 8000 cells/μL), and lymphocytes constitute the predominant leukocyte population compared to other domestic animals, in which segmented neutrophils are the dominant circulating leukocyte type [37].

### 2.3. Thrombocytes

Thrombocytes are cytoplasmic fragments of megakaryocyte lineage that play essential roles in maintaining vascular integrity, regulating hemostasis, and forming the primary hemostatic plug at sites of vascular injury [39,40]. Thrombocyte indices generated by automated hematology analyzers include (i) the platelet count (PLT), defined as the total number of platelets per unit volume of whole blood; (ii) the mean platelet volume (MPV), representing the average size of platelets; (iii) the platelet distribution width (PDW), indicating the variability in platelet volume; (iv) thrombocrit or plateletcrit (PCT), corresponding to the percentage of blood volume occupied by platelets; and (v) the platelet large cell count (P-LCC) and ratio (P-LCR), which quantify the absolute number and proportion of platelets with a volume greater than 12 fL, respectively [41,42]. Sheep thrombocytes are discoid with a smooth surface and occasional shallow surface depressions. Their mean diameter is approximately 2.6 μm—smaller than that in most domestic species, except goats. Despite their relatively small size, sheep exhibit a higher PLT than other common domestic animals [38].

## 3. Reference Intervals

Reference intervals represent the central 95% of values derived from apparently healthy animals and form the basis for interpreting hematological results. Ideally, reference intervals should be established using animals that share similar key characteristics with the target population (e.g., age, breed, sex, diet); homogenous populations tend to have narrower and more reliable reference intervals [14]. Variations in hematological parameters may be observed among clinically healthy animals, attributed to various physiological factors, such as age, breed, sex, diet, and stage of production, while procedural factors including animal restraint methods, blood collection techniques, and laboratory analytical procedures may also affect the results. Additionally, differences in analytical methods and population characteristics may lead to discrepancies in reference intervals among laboratories [20]. Table 2 illustrates reference intervals for hematological parameters in sheep across different population categories (general adult sheep, age- and breed- specific data) and analyzers. Notably, there are variations between analyzers in terms of the parameters that each analyzer reports, as well as in the upper and lower reference limits. Moreover, there are noticeable differences between age groups, with adult sheep showcasing higher upper limits for most of the erythrocyte indices, and between breed groups, with Merino lambs demonstrating higher upper limits for PLT. These variations highlight the importance of considering both population- and analyzer-specific reference intervals when interpreting results in sheep.

## 4. Physiological Factors Affecting Hematological Parameters

### 4.1. Blood Collection, Handling, and Analysis and Their Associations with Hematological Parameters

Blood collection in sheep is typically performed from the jugular vein using a syringe or evacuated tubes containing an anticoagulant such as ethylenediaminetetraacetic acid (EDTA), commonly in the form of dipotassium (K_2_EDTA) or tripotassium (K_3_EDTA) EDTA, which is the preferred anticoagulant for complete blood count analysis. For adult sheep, an 18–20-gauge, 1.5–2.0-inch-length needle is recommended, whereas a 22-gauge needle is more suitable for neonates. During venipuncture, animals should be calm and properly restrained either in a standing position or tipped with the head turned away [43]. Induced stress due to improper animal handling may cause the demargination of granulocytes, resulting in an artifactual increase in WBC [48]. Proper blood collection technique is essential, as traumatic venipuncture can lead to thrombocyte activation and clumping, causing a falsely decreased PLT [49].

Collection tubes must be filled to the appropriate volume to maintain the correct blood-to-anti-coagulant ratio and should be gently inverted several times to ensure adequate mixing [43]. Excess EDTA may cause erythrocyte shrinkage, resulting in a decreased PCV, and the appearance of echinocytes on blood smears, complicating leukocyte identification. Conversely, insufficient anticoagulant may lead to thrombocyte degranulation, clumping, aggregation, activation of the coagulation cascade, and visible fibrin formation [48].

Both the time interval between collection and analysis and the ambient temperature can influence hematological parameters [49]. Ideally, samples should be analyzed shortly after collection and blood smears performed immediately, since prolonged contact of blood with EDTA can alter the leukocyte morphology and cause the detachment of erythrocyte parasites [43]. Refrigeration at 4 °C ensures acceptable accuracy for complete blood count measurements for up to 24 h [50]. However, when thrombocyte evaluation is critical, blood samples should be analyzed within 6 h post-collection [39]. Storage at room temperature is not recommended, as it may result in a decreased WBC and promotes the formation of “smear cells” [48].

### 4.2. Animal-Related Factors

#### 4.2.1. Age

Age is one of the most influential intrinsic factors affecting hematological parameters in sheep. Ullrey et al. [51,52] investigated age-related variations in erythrocytes and leukocytes from birth to maturity in 316 purebred Hampshire, Shropshire, and Suffolk sheep. The red blood cell count decreased from 11.1 million cells/mm^3^ at birth to 8.8 million cells/mm^3^ at 8 days of age, followed by a progressive rise that peaked at 13 million cells/mm^3^ at 3 months. Corresponding fluctuations in HCT and HGB mirrored these trends; the highest values were recorded at birth (41.9% and 12.9 g/100 mL, respectively), whereas the lowest occurred at 14 days of age (27.2% and 8.9 gm/100 mL, respectively). In addition, the highest values of MCV and MCH were observed at the first day of life and gradually declined until approximately 5 months of age, while the MCHC was lowest at birth and increased thereafter. Similarly, Bornez et al. [53] reported that 70-day-old Manchega Spanish male lambs had significantly higher RBC, HGB, and HCT values and lower MCV and RDW values than 30-day-old lambs under on-farm conditions.

Pronounced differences between lambs and adult sheep have also been documented. Panousis et al. [24] examined the associations between age and hematological parameters in Chios sheep and observed that lambs aged 3 to 6 months had significantly higher RBC values than lactating ewes older than 1 year and higher HCT and HGB values than lactating ewes aged between 1 and 3 years. Similar patterns have been observed in Karakul sheep, where animals under 1 year old displayed significantly higher RBC and HGB values than older ones, possibly reflecting the elevated metabolic activity of younger animals [54]. In Moranda Nova sheep, higher RBC values were found in animals younger than 6 months compared with those aged between 6 and 12 months and those older than 1 year, although only the MCV differed significantly between the youngest and oldest groups [23]. Increased RBC and HGB values in lambs have been attributed to intensified erythropoiesis during late fetal development and the presence of fetal hemoglobin during early postnatal life [55]. Moreover, lambs consistently exhibit lower PLTs than adult animals [24], a pattern also reported in other ruminant species [56].

Individual leukocyte populations also vary significantly with age. During the post-natal period, neutrophils represent the predominant leukocyte type; however, by approximately 3–4 months of age, lymphocytes become dominant leukocyte type, yielding a neutrophil-to-lymphocyte ratio of about 0.5, which is the norm in healthy adult sheep [38]. In Hampshire, Shropshire, and Suffolk lambs, neutrophils increased from 34% at birth to 52% at 12 h of age, followed by a steady decline to 16% by one year of age, whereas lymphocytes displayed an inverse trajectory. Monocyte counts followed the same pattern observed for lymphocytes, while basophils remained a consistently minor leukocyte component across all ages [51]. Similar age-related trends have been reported in Chios sheep, where neutrophil proportions decreased with age and were the highest in lambs, whereas eosinophil counts were significantly lower in lambs than in lactating ewes older than 1 year [24].

The total WBC also exhibits marked age-dependent fluctuations. Ullrey et al. [51] reported the lowest WBC at birth (3.03 × 10^9^/L), which doubled within the first 12 h of life (6.16 × 10^9^/L), before declining at 48 h (4.26 × 10^9^/L). Subsequently, the WBC increased progressively, reaching a peak at 3 months of age (9.53 × 10^9^/L). In Chios sheep, the WBC was significantly lower in >3-year-old lactating ewes compared with lambs aged 3–6 months [24]. Furthermore, Korelidou et al. [57] demonstrated that age significantly influenced a broad range of hematological parameters, including the WBC, lymphocytes, monocytes, proportion of granulocytes and lymphocytes, RBC, HGB, MCV, MCH, PLT, MPV, PCT, and P-LCC, in healthy lactating Chios, Lesvos, and Lacaune ewes.

#### 4.2.2. Production Stage, Breed, and Sex

In addition to age-related changes, hematological parameters are also markedly influenced by the production stage. A comparative study of Barbados Belly and West African Dwarf sheep across different production stages indicated that dry non-pregnant ewes, followed by dry pregnant sheep, had significantly lower HGB, PCV, and neutrophil counts, as well as higher eosinophil counts, than growing lambs and lactating ewes [58]. Contrarily, dry pregnant Chios ewes exhibited significantly lower eosinophil counts and higher monocyte counts compared with lactating ewes [24]. Ullrey et al. [51,52] described hematological fluctuations during gestation in Hampshire, Shropshire, and Suffolk ewes; the lowest RBC, HCT, and HGB values occurred at approximately 4.5 months of gestation, followed by an increase at parturition and a subsequent decline 2 weeks post-partum. Leukocyte counts tended to increase during gestation, peaking at parturition before decreasing again two weeks later. Throughout gestation, lymphocytes were the dominant leukocyte type (57–61%), whereas segmented neutrophils were the dominant leukocyte type (60%) at parturition.

Korelidou et al. [57] found that the stage of lactation significantly affected a range of hematological parameters, including the monocyte proportion, HCT, MCV, MCH, MCHC, red cell distribution width—coefficient of variation (RDW-CV), red cell distribution width—standard deviation (RDW-SD), PLT, MPV, PDW, PCT, P-LCC, and P-LCR. The latter study also revealed breed-related variations in WBC, granulocytes, monocytes, and their proportions, as well as RBC, HGB, HCT, MCV, MCHC, RDW-SD, PLT, MPV, PDW, PCT, P-LCC, and P-LCR. Earlier, Kalogianni et al. [59] reported significantly higher monocyte counts, P-LCR, and MPV and a lower RDW-CV in lactating Chios ewes compared with Lacaune ewes at the end of lactation.

Breed-associated variation in hematological traits has been widely documented. For instance, non-lactating, non-pregnant Bergamaska ewes had significantly lower values of PCV, RBC, and MCV compared to Santes Ines and crossbred Santa Ines × Bergamaska ewes [60]. In another study, no significant differences in hematological parameters were found between Santa Ines and Moranda Nova sheep, yet Soinga sheep displayed significantly higher RBC and HCT values and lower MCVs compared with Moranda Nova sheep [22]. Additionally, Chios rams exhibited significantly lower RBC, HGB, HCT, and RDW values alongside higher WBC, lymphocyte, monocyte, and eosinophil counts compared to Florina rams [21].

Sex-related differences in hematological parameters have been reported in many species [48], reflecting hormonal influences and differences in reproductive physiology between males and females. In numerous mammals, RBC values tend to be higher in males due to the erythropoietic effects of androgens, particularly testosterone [61,62]. However, relevant studies specifically evaluating sex-related hematological variations in sheep remain limited. For example, Fadare et al. [63] reported that no significant differences in PCV, RBC, and WBC were observed between male and female West African Dwarf sheep.

### 4.3. Management- and Breeding Environment-Related Factors

#### 4.3.1. Altitude

Management practices and breeding environments may also influence hematological parameters. Variations in HCT, RBC, and HGB have been observed in animals reared at different geographical locations and altitudes, with sheep grazing at high altitudes exhibiting increased values of these parameters. This response is particularly attributed to the increased production and release of erythropoietin as an adaptive mechanism to compensate for reduced oxygen availability at high altitudes [30]. Šoch et al. [64] compared the hematological profiles of sheep reared at different altitudes and confirmed that the HGB and HCT values were significantly higher in sheep reared at 950 m compared with those reared at 800 m and 550 m. The same study also reported lower WBC values in sheep reared at higher altitudes; interestingly, the highest proportion of eosinophils was observed at 550 m, whereas monocyte counts did not differ significantly among the studied altitude groups.

#### 4.3.2. Season

Seasonal variation in hematological parameters, including RBC, has also been documented, although the magnitude of these changes depends on the breed, geographical location, and study design [38]. For example, the HGB and HCT values in Sumavian, Merinolandschaf, and Charolais sheep were significantly higher in the fall than in the spring, while WBC values were lower during the same period [64]. Similarly, Merino and East Friesian sheep exhibited significantly higher HGB values and lower MCHC and RDW-CV values in December than in May [65]. Conversely, Baumgartner et al. [54] reported that the PCV and HGB were significantly higher during the summer than in winter.

Increases in erythrocyte count or mass may reflect hemoconcentration resulting from dehydration [30], a common consequence of heat stress. Turini et al. [66] evaluated the effects of pasture management and heat stress in 60 sheep and found that the RBC, HCT, and HGB values were significantly higher in animals grazing on pastures without access to shade or water compared to those provided with shelter and water during periods of severe heat stress. In another study, Fadare et al. [63] observed that black-coated West African Dwarf sheep, which were more affected by heat stress, displayed lower WBC and higher RBC values than light-coated sheep. The same study also reported seasonal variation in hematological indices, with the RBC being significantly higher during the late dry season (April–June) and WBC being higher during the early dry season (January–March). According to the authors, these patterns likely reflect physiological responses to the heat load and an increased demand for oxygen transport during panting.

Changes in hematological parameters may arise in situations of stress, fear, excitement, or intense exercise due to the actions of epinephrine and cortisol. Increased cardiac output under these conditions can lead to physiological leukocytosis, characterized by neutrophilia, lymphopenia, and eosinopenia (with the latter being less apparent in ruminants, where reference intervals for eosinophils often begin at zero) and occasionally monocytosis [38,39].

#### 4.3.3. Time of Day

With regard to the time of collection, McManus et al. [60] collected blood samples from 50 non-lactating, non-pregnant Santa Ines, Bergamaska, and crossbred Santa Ines × Bergamaska ewes at two different time points during the day, namely 6 a.m. and 2 p.m., and observed higher WBC values in the afternoon, possibly reflecting increased blood pressure and heart rate. However, no specific diurnal pattern was identified for other hematological parameters. Bórnez et al. [53] evaluated the effects of the sampling time (on farm, after transport, and after lairage) on hematological parameters in 30- and 70-day-old Manchega lambs. No significant differences were observed between on-farm and post-transport blood samples. After lairage, however, the RBC, HGB, and HCT increased significantly in 70-day-old lambs, possibly due to catecholamine-induced splenic contraction, whereas, in 30-day-old lambs, the HGB, MCH, MCHC, and WBC decreased.

#### 4.3.4. Diet and Nutritional Status

Animal diet is associated with nutrient availability, digestibility, and absorption, thereby affecting hematological parameters. To minimize any effect of diet or water on hematological parameters, in many studies, blood samples are collected in the morning before feeding [46]. Essential trace minerals such as iron, cobalt, copper, and selenium are critical for animal metabolism and the formation and normal production of blood components. Deficiencies or toxicities due to these minerals have been associated with alterations in erythrocyte indices, with the most common being anemia [67]. In addition, several studies have examined the effects of diet ingredients on hematological profiles; for instance, the inclusion of urea-treated rumen digesta pellets in Djallonke rams did not alter hematological parameters [68]. Similarly, hematological parameters were not affected in Sarda ewes after the replacement of soybean hulls with cocoa husks in the diet, with the exception of the basophil count, which was significantly lower in the group fed with cocoa husks [69].

Nutritional status is also linked to hematological profiles. In clinically healthy lactating Chios, Lesvos, and Lacaune ewes, the body condition score (BCS) was significantly associated with the monocyte proportion, RBC, HGB, HCT, RDW-CV, and RDW-SD [57]. Likewise, in clinically healthy Pelibuey ewes, a statistically significant positive correlation was observed between BCS and RBC (r = 0.35, *p* < 0.05), HCT (r = 0.39, *p* < 0.05), RDW-SD (r = 0.45, *p* < 0.05), and RDW-CV (r = 0.48, *p* < 0.001) [70]. In Ile de France sheep, animals with a BCS of 3.0–3.5 exhibited significantly higher values of RBC, HCT, and HGB compared to those with a BCS of 2.0–2.75 [71].

## 5. Hematological Parameters and Their Associations with Health Conditions

### 5.1. Erythrocytes

**Figure 1:**
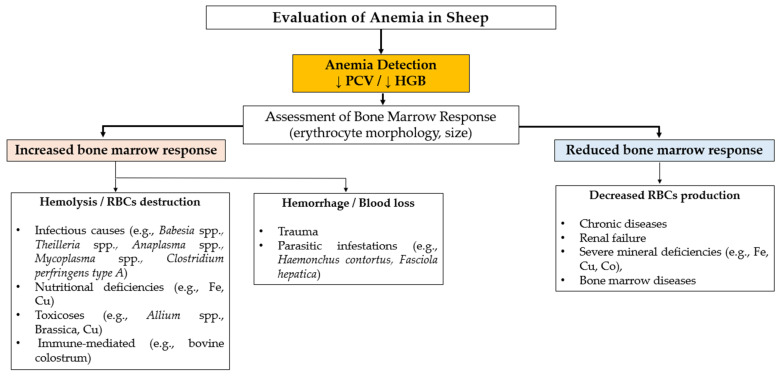
Schematic representation of anemia in sheep. Cobalt (Co), copper (Cu), hemoglobin (HGB), iron (Fe), red blood cells (RBCs), packed cell volume (PCV).

Anemia is the most common health condition associated with alterations in the erythrogram and is defined by a reduction in the RBC and/or HBG concentration. This reduction impairs the oxygen-carrying capacity of the blood, resulting in decreased tissue oxygenation. Anemia usually reflects an underlying health disorder involving increased erythrocyte destruction, blood loss due to hemorrhage, or reduced RBC production [43]. To aid in understanding the underlying cause, anemia is sometimes described as regenerative or non-regenerative, although circulating reticulocytes are rarely present in large numbers in sheep. Regenerative anemia, typically associated with blood loss or RBC destruction, is often inferred from indirect signs of bone marrow response (e.g., erythroblasts, polychromasia), whereas non-regenerative anemia often reflects defective or insufficient erythropoiesis [72] (Figure 1).

Anemia may also be classified according to the erythrocyte size and HGB content, as indicated by the MCV and MCHC, respectively. Based on these indices, anemia is described as microcytic, normocytic, or macrocytic and as hypochromic, normochromic, or hyperchromic when the MCV and MCHC are decreased, normal, and increased, respectively [72]. Morphological abnormalities may accompany anemia and are associated with regenerative responses, immune-mediated and oxidative damage, membranic defects, metabolic disorders, or mechanical fragmentation. In any case, morphological findings should be interpreted along with other qualitative and quantitative data from the complete blood count [31].

Increases in PCV or HCT (erythrocytosis/polycythemia) are most commonly associated with dehydration and a decreased plasma volume and less frequently with conditions causing hypoxemia (e.g., chronic pulmonary disease) or neoplastic processes (e.g., cholangiocellular carcinoma) [28,38].

#### 5.1.1. Erythrocyte Loss

Erythrocyte loss is associated with hemorrhage, which may be internal, occurring in a body cavity, or external [38,39]. Acute blood loss typically triggers a regenerative response; however, chronic external hemorrhage is often accompanied by a weaker regenerative response and the development of iron deficiency anemia due to the prolonged loss of erythrocyte components and iron. The progressive depletion of iron reserves may eventually lead to non-regenerative anemia [39].

The most common parasites associated with blood loss anemia in sheep are *Haemonchus contortus* and *Fasciola hepatica* [73]. The severity of anemia depends on the infection burden, the developmental stage of the larvae, and the age and body weight of the affected animal. Hyperacute hemonchosis, although uncommon, is characterized by extremely heavy burdens of *Haemonchus contortus*, leading the affected sheep to lose up to 18–54 g of HGB per day [74]. In acute hemonchosis, blood loss is less severe, and anemia develops more gradually; PCV decreases progressively, and compensatory erythropoiesis becomes evident within the first 14 days of infection [75].

Natural infection with *Haemonchus contortus* has been associated with microcytic, hypochromic anemia resulting from abomasal hemorrhage and chronic blood loss [76]. In an experimental study involving Santa Ines male lambs infected with both *Haemonchus contortus* and *Trichostrongylus colubriformis*, the RBC, PCV, and HGB values began to decline significantly at 15 days post-infection, whereas the MCV and MCHC did not differ significantly between infected and healthy animals [77].

Organically farmed German Merino sheep naturally infected with *Fasciola hepatica* exhibited significantly lower RBC, HGB, PCV, and MCHC values and higher MCV values compared with non-infected ones [78]. A similar pattern has been observed in animals with chronic subclinical fasciolosis [79]. In native Balady sheep, *Fasciola hepatica* infection has been associated with significantly lower RBC, HGB, and PCV values by 38.4%, 48.4%, and 42.4%, respectively [80].

Acute blood loss has also been linked to oxidative stress; De Sousa et al. [81] experimentally induced acute hemorrhage via phlebotomy in 18 healthy sheep and found that the RBC, HCT, and HGB began to decline within 30 min post-phlebotomy, reaching their lowest values at 24 h.

#### 5.1.2. Erythrocyte Destruction

Erythrocyte destruction (hemolysis) may be physiological or pathological and involves the removal of old, damaged, parasitized, or morphologically abnormal erythrocytes from circulation, either by the splenic macrophages or within the vasculature [39]. It is usually attributed to RBC parasites, infectious or toxic agents, nutritional deficiencies, and toxicities, as well as immune-mediated processes or neoplastic conditions [82], which are rare in sheep. Hemolytic anemia is initially normocytic and normochromic, but, with time, it may evolve into macrocytic hypochromic or macrocytic normochromic anemia [30].

Infection with *Babesia* spp., *Theilleria* spp., *Anaplasma* spp., *Trypanosoma* spp., or *Mycoplasma* spp. has been associated with hemolytic anemia in sheep [73]. Natural infection with *Theilleria* spp. results in a significant decrease in RBC, HGB, and PCV, largely due to increased oxidative stress on erythrocytes. The same study reported a negative correlation between the parasitemia level and RBC (r = −0.36, *p* < 0.01), HGB (r = −0.77, *p* < 0.01), and PCV (r = −0.78, *p* < 0.01) [83]. Similarly, sheep and goats naturally infected with *Babesia ovis* exhibited significantly lower RBC, PCV, HGB, MCV, and MCHC values, which decreased further as parasitemia increased. In such cases, blood smear evaluation indicates microcytic, hypochromic, and hemolytic anemia [84]. In contrast, another study involving 97 sheep reported normocytic, normochromic anemia associated with *Babesia ovis* infection [85]. Chronic *Babesia ovis* infection has also been associated with macrocytosis and hyperchromasia. Infection with *Babesia lengau* results in anisocytosis, polychromasia, and the presence of Heinz bodies and Howell–Jolly bodies within erythrocytes [86].

*Anaplasma* spp. and *Mycoplasma* spp. induce spleen-mediated extravascular hemolysis [39]. Ovine anaplasmosis, primarily caused by *Anaplasma ovis*, has been associated with normocytic, normochromic anemia [87]. PCR-positive lambs exhibit severe anemia, and animals with icteric carcasses show significantly lower RBC, HCT, and HGB values compared with unaffected animals [88].

*Mycoplasma ovis* is a hemotropic parasitic bacterium that adheres to erythrocytes and induces hemolytic anemia. The severity of anemia varies with age, nutritional and immunological status, and concurrent infections. Acute mycoplasmosis can lead to severe hemolytic anemia and high mortality, particularly in young animals, whereas chronic infections are associated with mild anemia [89]. Finally, *Trypanosoma* spp. may also cause anemia, with erythrocyte destruction occurring in later stages of infection due to increased phagocytic activity [43].

Bacterial toxins produced by *Leptospira* spp. and *Clostridium perfingens* can also induce hemolytic anemia [90]. *Clostridium perfingens* type A, also known as the cause of “yellow lamb disease”, may lead to severe anemia, with PCV values dropping to as low as 4.0%, often resulting in fatalities in affected lambs [91].

With regard to nutrition, anemia may result from iron or copper deficiency. Iron plays an essential role in numerous metabolic processes, including erythropoiesis. Iron deficiency may appear in two principal forms: absolute iron deficiency, which is primarily caused by chronic hemorrhage and less frequently by insufficient gastrointestinal absorption or iron-poor diets, and functional iron deficiency, which is associated with inflammation. In the latter case, hepcidin produced during inflammation binds to ferroportin, thereby restricting the release and availability of iron reserves for hemoglobin production. Both forms of iron deficiency are characterized by decreased RBC, HCT, and HGB values. Absolute iron deficiency typically results in microcytic, hypochromic anemia and is usually accompanied by an increased RDW. Conversely, functional iron deficiency is associated with normocytic, normochromic anemia [92]. Notably, iron deficiency anemia is predominantly observed in lambs rather than in adult sheep due to their higher iron requirements [93].

Copper deficiency may develop in animals consuming forage grown on copper-deficient soils or diets high in sulfate. Affected animals may develop microcytic, hypochromic anemia [92]. Deficiencies in copper and selenium have also been linked to the formation of Heinz bodies within erythrocytes [94].

Anemia in sheep may also result from oxidative injury or plant-associated toxicoses. Some plant species contain bioactive compounds, such as alkaloids, glucosinolates, glycosides, oxalates, and coumarins, which can induce hematologic disturbances following ingestion [95]. For instance, plants of the Allium genus have sulfur-containing compounds, and their consumption by sheep has been associated with the development of hemolytic anemia [96]. In one reported case, sheep grazing for one month in an onion field exhibited an approximately 25% reduction in PCV, as well as decreased RBC and HGB concentrations. Hematologic evaluation further revealed polychromasia and mild leukocytosis, while blood smears demonstrated Heinz body formation within erythrocytes [97].

Additionally, plants of the Brassica genus (e.g., kale, stubble, turnips, and root crops) contain toxic compounds such as S-methyl-cysteine sulfoxide, and their ingestion may lead to macrocytic, normochromic anemia, often with Heinz–Ehrlich bodies present in erythrocytes [98]. Bracken fern (*Pteridium aquilinum*) also contains toxic compounds such as pruinose, ptaquiloside, and thiaminases, which may lead to anemia. Although bracken fern toxicosis primarily affects young cattle, sheep can also be affected [95].

Copper toxicosis in sheep may result in hemolytic anemia due to oxidative damage to erythrocytes, particularly in chronically overfed animals subjected to stressful conditions [43]. Affected red blood cells typically exhibit anisocytosis, while large numbers of normoblasts may be observed in blood smears [98]. Macrocytosis, hypochromasia, and basophilic stippling have also been reported in some cases [99].

Immune-mediated erythrocyte destruction has been associated with the ingestion of bovine colostrum by neonatal lambs. Clinical signs of neonatal anemia emerge when the PCV declines to approximately 10%, and mortality may occur when values fall to 3.0–4.0% [100].

#### 5.1.3. Decreased Production of Erythrocytes

Inadequate erythrocyte production in bone marrow most commonly stems from extrinsic conditions that secondarily impair marrow function and less frequently from intrinsic marrow disorders, which are typically associated with pancytopenia (leukopenia and thrombocytopenia) [39]. Anemia with poor to absent regeneration is usually normocytic and normochromic [28] and is most often attributed to chronic diseases (e.g., caseous lymphadenitis), also known as anemia of chronic disease, and less frequently to renal failure [38]. The pathogenesis of anemia of chronic diseases is multifactorial and involves disturbances in iron homeostasis, impaired proliferation of erythroid progenitor cells, altered production of erythropoietin, and a shortened erythrocyte lifespan. In contrast, anemia associated with renal failure is primarily caused by reduced erythropoietin production by diseased kidneys [101] and may be more severe than anemia of chronic disease [102].

Anemia with mild regeneration may occasionally be observed in pregnant ewes and in animals with marked deficiencies in essential minerals, such as iron, copper, or cobalt [39,43]. Iron deficiency typically induces regenerative anemia; however, it can evolve to a non-regenerative form in advanced stages [101]. Chronic copper deficiency disrupts intracellular iron mobilization and heme synthesis [39], while cobalt deficiency has been linked to decreased hemoglobin concentrations, as reported in lambs affected by white liver disease [103].

### 5.2. Leukocytes

Changes in total or differential leukocyte counts, as reflected in leukograms, can provide valuable insights into the underlying nature of disease processes [104] (Table 3). Alterations in specific leukocyte types are often more informative than the WBC alone, which may remain within reference intervals despite significant inflammatory, stress-related, or neoplastic responses [33]. Marked leukocytosis (an increase in WBC above reference intervals) is relatively uncommon in sheep because of the limited storage of neutrophils within the bone marrow; however, when it occurs, it is usually associated with chronic inflammatory conditions [19]. Leukopenia (a decrease in the total WBC below reference intervals), on the other hand, is commonly observed in viral infections, metabolic disorders, and the early stages of severe bacterial diseases [33].

#### 5.2.1. Polymorphonuclear Cells

Neutrophils are key mediators of inflammation, functioning through chemotactic migration to sites of tissue injury or inflammation and the phagocytosis of pathogens and foreign substances [105]. The neutrophil-to-lymphocyte (N:L) ratio often provides more relevant diagnostic information than the absolute count of either cell type alone [102]. Neutrophilia (increase in neutrophil count above reference intervals) may arise from three main leukogram patterns: an inflammatory leukogram, a stress leukogram, or an excitement-related (physiologic) response [39].

An inflammatory leukogram is characterized by an increased N:L ratio accompanied by the presence of immature neutrophils (bands) and occasionally other immature forms such as metamyelocytes. In contrast, an elevated ratio of N:L exceeding 2:1 in the absence of band neutrophils is indicative of a stress leukogram [102], which commonly results from non-inflammatory conditions or following corticosteroid administration [38]. Neutrophilia is frequently associated with Gram-positive, subacute, or chronic bacterial infections [43], as well as with immune-mediated hemolytic anemia [104], which is, however, very rare in sheep. In cases of more severe inflammation, the acute phase is characterized by toxic changes and immature forms of neutrophils (band cells, metamyelocytes, or myelocytes). The persistence of these findings over time is generally associated with a poor prognosis [102]. Table 3 presents several diseases and changes in hematological parameters. Briefly, several bacterial diseases in sheep, such as enteric infections due to *Clostridium perfrigens*, blackleg, black disease, and malignant edema and braxy have been associated with neutrophilic leukocytosis with a left shift [43,106]. Neutrophilia has been also reported in conditions such as acute renal disease [102,107], pituitary abscess syndrome [108], bacillary hemoglobinuria [43], pericarditis and vegetative endocarditis [109], and mastitis [110,111].

Severe acute inflammation caused by Gram-negative bacterial infections may lead to a transient decrease in neutrophils (neutropenia), often accompanied by a left shift (increased immature forms) [43]. Neutropenia typically develops within 24 to 48 h after the onset of severe inflammation because of the limited circulating neutrophil pool in sheep [102] and generally resolves within 3–4 days as the peracute phase of the disease subsides [43]. Neutropenia has been also observed in sheep with tick-borne disease (*Anaplasma phagocytophilum*) [112], septic shock [109], and certain toxic conditions, such as stachybotritoxicosis [113].

Eosinophils are primarily responsible for host defense against parasitic infections and contribute to the pathogenesis of type 1 hypersensitivity allergic reactions [33,37]. For instance, infections due to *Fasciola hepatica, Fasciola magna*, and *Osteria ovis* have been found to be associated with eosinophilia [114,115]. Eosinophils exhibit limited effectiveness in combating bacterial and viral pathogens. A decrease in eosinophil numbers below reference intervals (eosinopenia) is generally of limited clinical significance and is most commonly associated with stress responses [37].

Circulating basophils are rarely observed in blood smears from healthy sheep. Basophilia (an increase in basophil counts above reference intervals) is generally of limited clinical significance [43]. However, it may occur concurrently with eosinophilia, in response to the release of inflammatory mediators, such as histamine [38].

#### 5.2.2. Mononuclear Cells

Lymphocytes comprise T-lymphocytes, which mediate cellular immunity (including immune regulation, cytotoxic responses, delayed-type hypersensitivity, and graft-versus-host reactions); B-lymphocytes, which are responsible for humoral immunity; and natural killer (NK) cells. Lymphocytosis (an increase in lymphocyte counts above reference intervals) is not common in ruminants but may occur secondary to chronic inflammatory processes (e.g., internal abscessation) or to non-chronic inflammatory conditions. Lymphocytosis has been observed in cases of coenuruses [37], infection with bovine leukemia virus [116], and peste de petites during the incubation period [117]. In contrast, lymphopenia (a decrease in lymphocyte counts below reference intervals) commonly develops as part of a stress response, with numerous diseases being capable of triggering such a response [38]. Measurement of the plasma fibrinogen concentration can assist in determining whether lymphopenia and neutrophilia reflect a stress response or inflammation. Particularly high fibrinogen concentrations, along with toxic changes and high counts of immature neutrophils, are indicative of inflammation [43].

Monocytes play several essential roles, including phagocytosis, antigen presentation to T-lymphocytes, and immunomodulation through the production of cytokines that regulate inflammation and hematopoiesis. Increased monocyte counts may serve as an indicator of stress or chronic inflammatory processes [38].

**Table 3:** Leukogram changes associated with various diseases.

Disease	Leukogram Change	Comments	Ref.
**Bacterial**			
Enteric infections (*Clostridium perfringens)*	Neutrophilic leukocytosis with left shift		[43]
Black disease (*Clostridium novyi* type B)	Neutrophilic leukocytosis with left shift		[43,106]
Blackleg (*Clostridium* spp.)	Neutrophilic leukocytosis with left shift	Possible ↓ RBC and WBC	[43]
Malignant edema and braxy (*Clostridium* spp.)	Neutrophilic leukocytosis with left shift	Possible ↓ RBC and WBC	[43]
Bighead (*Clostridium novyi type A*)	Neutrophilic leukocytosis with degenerative left shift		[43]
Acute renal disease (*Leptospira* spp.)	↑ WBC and neutrophilia	Hemolytic anemia	[102,107]
Pituitary abscess syndrome	Leukocytosis and neutrophilia		[108]
Bacillary hemoglobinuria (*Clostridium hemolyticum*)	Leukocytosis with mature neutrophilia, degenerative left shift		[43]
Pericarditis	Leukocytosis, absolute neutrophilia or lymphopenia	Mild anemia	[109]
Vegetative endocarditis	Neutrophilia (with or without left shift)	Non-regenerative anemia	[109]
Subclinical mastitis (*Staphylococcus* spp., *Corynebacterium* spp.)	↑ WBC, neutrophils, lymphocytes		[110]
Clinical mastitis (*Staphylococcus aureus*, *Escherichia coli*)	↑ WBC and neutrophils, ↓ lymphocytes		[111]
Pasteurellosis (*Pasteurella multocida*)	↑ Immature neutrophils		[43]
Gram-negative sepsis	Panleukopenia → ↑ immature neutrophils → ↑ mature neutrophils and restoration of lymphocyte count		[43]
Neonatal diarrhea (*Salmonella* spp.)	Leukocytosis or leukopenia		[118]
Osteomyelitis (*Corynebacterium* spp., *Actinomyces pyogenes*, *Escherichia coli*)	Leukocytosis or leukopenia		[113]
Tick-borne fever (*Anaplasma phagocytophilum*)	Neutropenia	Thrombocytopenia	[112]
Caseous lymphadenitis (*Corynebacterium pseudotuberculosis*)	↑ Monocytes		[119]
**Viral**			
Bluetongue virus	Leukopenia with possible lymphopenia	Thrombocytosis	[43,120,121]
Peste de petites virus	Lymphocytosis → leukopenia (lymphopenia)		[43,117]
Bovine leukemia virus	Lymphocytosis		[116]
Ovine progressive pneumonia (maedi visna virus)	Leukocytosis	Hypochromic anemia	[122]
**Parasitic**			
Coenuruses (*Coenurus cerebralis*)	Lymphocytosis		[37]
Faschiolosis (*Fasciola hepatica, magna*)	Eosinophilia	Anemia	[114]
Oestrosis (*Osteria ovis*)	Eosinophilia		[115]
**Metabolic**			
Pregnancy toxemia	Neutropenia/or normal, eosinophilia, lymphocytosis		[118]
**Toxic**			
Stachybotrytoxicosis	Neutropenia, lymphopenia	Thrombocytopenia	[113,123]
Bracken fern consumption	↓WBC	↓ Thrombocytes	[124]
**Systemic**			
Septic/maldistributive shock	Neutropenia, immature neutrophils	Thrombocytopenia	[109]
**Neoplastic**			
Adenocarcinoma of pituitary gland	Leukocytosis, mature neutrophilia	↑ PCV, RBC, HGB	[108,125]

Red blood cell count (RBC), hemoglobin (HGB), packed cell volume (PCV), white blood cell count (WBC), increase (↑), decrease (↓), lead (→)

### 5.3. Thrombocytes

Thrombocytes generally constitute the primary defense against hemorrhage and play essential roles in thrombotic, inflammatory, and neoplastic processes (rare in sheep), functioning through adhesion, aggregation, granule secretion, retraction, microvesiculation, and the expression of procoagulant activity [126]. In addition to hemostasis, thrombocytes contribute to host defense during infection by directly interacting with leukocytes and endothelial cells, secreting cytokines and chemokines, and contributing to the activation of complement systems [127]. A decrease in circulating thrombocytes below reference intervals (thrombocytopenia) may arise from (i) platelet sequestration; (ii) decreased or defective platelet production due to bone marrow disorders, typically resulting in moderate-to-severe thrombocytopenia; and (iii) increased platelet consumption or destruction, as observed in severe trauma, acute external hemorrhage, or exchange blood transfusion, which most commonly leads to mild-to-moderate thrombocytopenia [41,127]. Plateletcrit is considered a good indicator of overall platelet functional capacity [41] and may serve as a predictor of thrombocytopenia [127]. However, PCT measurements can be prone to errors; thus, PLT is often best confirmed using a stained blood smear. Among other platelet indices, increased MPV and PDW are associated with the presence of large or giant platelets, which indicate a platelet regenerative response, and greater variations in platelet volume, respectively [41].

In sheep, severe thrombocytopenia has been associated with conjunctival hemorrhages following trauma [124]. A reduction in platelet number has also been documented in experimental infections with *Theileiria lestoquardi* [128] and observed alongside neutropenia in infections caused by *Anaplasma phagocytophilum* and toxins of *Stachybotrys* spp. [123,129]. Thrombocytopenia has also been observed in anemic sheep infected with *Babesia ovis* in a study that concluded that a reduced PLT and PCT suggest either impaired platelet production or increased consumption, while an elevated MPV indicated active bone marrow function to compensate for the decreased platelet level [85]. Thrombocytopenia has also been described in bluetongue virus infections, where it has been attributed to consumptive coagulopathy secondary to vasculitis [39,120], as well as in sheep seropositive for maedi visna virus [130]. In contrast, mixed infections with *Haemonchus contortus* and *Trichostrongylus colubriformis* have been associated with significantly increased platelet counts, likely reflecting a response to abomasal hemorrhage induced by *Haemonchus contortus* [77]. In ruminants, an increase in PLT above the reference interval (thrombocytosis) [131] is usually secondary and most commonly associated with exercise, stress, or inflammatory processes [39].

## 6. Conclusions

Modern hematological analyzers have advanced to incorporate species-specific analytical capabilities and provide standardized and quantitative results rapidly and at a lower cost compared to conventional laboratory methods. Hematological parameters serve as valuable tools for screening and monitoring, guiding the prioritization of further diagnostic approaches, rather than functioning as a standalone diagnostic tool. Indeed, the combination of hematological parameters with animal history, clinical signs, and physical examination offers valuable insights into animal health and welfare and, in turn, contributes to improved management practices and herd health-level surveillance. In particular, hematological assessments can be useful in the early detection of infectious, inflammatory, parasitic, and haemopoietic disorders at the individual animal level. Moreover, longitudinal monitoring of hematological profiles at the herd level may facilitate the detection of subclinical diseases, the evaluation of health trends, and the timely implementation of management interventions before clinical outbreaks occur.

However, many hematological alterations are non-specific and require careful interpretation. The interpretation of hematological parameters in sheep is complicated by the absence of case-specific standardized reference intervals and by the wide range of physiological and pathological factors affecting these parameters. Pre-analytical and analytical factors including sampling techniques, anticoagulant use, sample storage, and the type of analyzer can also affect results. Consequently, the interpretation of hematological parameters obtained from complete blood count tests should be performed within the appropriate clinical and biological context, taking into consideration natural variability and non-pathological factors that may influence these values. Currently, most available reference intervals are derived from heterogenous populations encompassing various ages, breeds, and physiological stages; thus, results should not be interpreted rigidly, and values outside reference intervals should be evaluated cautiously, as they do not necessarily indicate the presence of disease. Within this framework, hematological parameters can be effectively integrated into routine screening and benchmarking activities at the animal and flock levels. Such an approach allows comparative evaluation among animals, including those closer to the upper and lower limits of the reference intervals, as well as across different time periods and management practices (e.g., deworming efficiency). A practical interpretative strategy involves using pattern recognition, evaluating groups of related parameters rather than isolated values, and monitoring temporal trends in hematological variation.

Moreover, hematological characterization for many sheep diseases remains limited, with much of the available literature dating back more than three decades. The development of age-, breed-, and physiological stage-specific hematological benchmarks would enhance the diagnostic accuracy and promote broader adoption as a routine component of sheep health management. Therefore, future research should prioritize large-scale studies encompassing various breeds, production systems, age groups, geographic locations, and health statuses to establish robust reference intervals. The harmonization of sampling protocols and validation of hematological analyzers will also be essential to improve data reliability and comparability across studies. Hematological parameters can complement production, welfare, and sensor-based monitoring systems, and, when combined with epidemiological data, they can enhance preventive health strategies. Thus, advances in sheep hematology will further support the use of hematological profiling as a modern and informative component of overall sheep health management practices.

## Figures and Tables

**Table 1:** Erythrocyte parameters.

Parameter	Definition
Red blood cell count (RBC)	Number of erythrocytes per unit volume of blood, typically expressed as ×10^6^/µL.
Hematocrit (HCT)	The proportion of blood volume occupied by erythrocytes, [RBC (10^6^/µL) × MCV (fL)]/10.
Packed cell volume (PCV)	The percentage of blood volume composed of erythrocytes (defined by microhematocrit centrifugation).
Mean corpuscular volume (MCV)	The average volume of individual erythrocytes, expressed in femtoliters (fL), [HCT (%) × 10]/RBC (10^6^/µL).
Mean corpuscular hemoglobin (MCH)	Average hemoglobin (HGB) content in a single erythrocyte, expressed in picograms (pg), [HGB (g/dL) × 10]/RBC (10^6^/µL)].
Mean corpuscular hemoglobin concentration (MCHC)	Average cell hemoglobin concentration within a given volume of erythrocytes, expressed in g/dL, [HGB (g/dL) × 100]/HCT (%)].

**Table 2:** Reference intervals for sheep hematological parameters from various population sources.

Parameter (Unit)	Adult Sheep [43]	VetScan Analyzer [44]	Mindray Analyzer [45]	Merino Lambs [46]	Florina and Chios Rams [47]
RBC (10^6^/μL)	9.0–17.5	9.0–15.8	6.5–15.2	9.2–13.0	9.2–13.7
HGB (g/dL)	9.0–15.8	9.0–15.0	6.8–14.5	10.5–13.7	9.5–14.5
HCT (%)	27.0–45.0	27.0–45.0	20.0–42.5	28.0–39.0	27.3–40.7
PCV (%)	na	na	na	30.0–43.0	na
MCV (fl)	28.0–40.0	28.0–40.0	25.0–41.0	28.0–35.0	25.4–34.1
MCH (pg)	na	8.0–12.0	8.0–12.3	na	9.0–11.9
MCHC (g/dL)	31.0–34.0	31.0–34.0	29.0–37.0	33.2–39.2	32.5–39.7
RDW-CV (%)	na	na	14.5–26.2	na	15.9–20.3
RDW-SD (fL)	na	na	17.0–32.0	na	na
WBC (10^9^/L)	4.0–12.0	4.0–12.0	5.1–15.8	5.1–15.9	6.1–14.2
Lymphocytes (10^9^/L)	2.0–9.0	2.5–7.5	2.0–7.8	2.1–10.2	2.8–9.3
Lymphocytes (%)	na	na	28.0–71.5	na	na
Neutrophils (10^9^/L)	1.5–9.0	0.7–7.3	na	0.8–6.3	1.4–7.1
Eosinophils (10^9^/L)	0.0–1.0	na	na	0.0–0.2	0.0–0.7
Eosinophils (%)	na	na	na	na	na
Monocytes (10^9^/L)	0.0–0.6	0.0–0.8	0.0–1.3	0.1–0.8	0.0–0.7
Monocytes (%)	na	na	0.0–9.5	na	na
Basophils (10^9^/L)	0.0–0.3	na	na	0.0–0.2	0.0–0.1
Granulocytes (10^9^/L)	na	na	1.3–7.6	na	na
Granulocytes (%)	na	na	21.5–68.0	na	na
PLT (10^3^/μL)	240.0–700.0	100.0–800.0	200.0–800.0	426.0–1142.0	229.0–823.0
MPV (fL)	na	na	3.5–6.8	na	4.1–9.9
PDW	na	na	12.0–17.5	na	na
PCT (mL/L)	na	na	1.0–4.2	na	1.6–4.9
P-LCC (10^9^/L)	na	na	30.0–260.0	na	na
P-LCR (%)	na	na	12.6–60.0	na	na

Red blood cell count (RBC), hemoglobin (HGB), hematocrit (HCT), packed cell volume (PCV), mean corpuscular volume (MCV), mean corpuscular hemoglobin (MCH), mean corpuscular hemoglobin concentration (MCHC), red cell distribution width—coefficient of variation (RDW-CV), red cell distribution width—standard deviation (RDW-SD) white blood cell count (WBC), platelet count (PLT), mean platelet volume (MPV), platelet distribution width (PDW), plateletcrit (PCT), platelet large cell count (P-LCC), platelet large cell ratio (P-LCR), not available (na). The values provided by VetScan and Mindray analyzers are generally for sheep without additional clarification.

## Data Availability

No new data were created or analyzed in this study. Data sharing is not applicable.

## Abbreviations

The following abbreviations are used in this manuscript:

BCS	Body condition score
EDTA	Ethylene-diamine-tetra-acetic acid
HCT	Hematocrit
HGB	Hemoglobin
MCH	Mean corpuscular hemoglobin
MCHC	Mean corpuscular hemoglobin concentration
MCV	Mean corpuscular volume
MPV	Mean platelet volume
N:L	Neutrophil-to-lymphocyte ratio
RBC	Red blood cell count
RBCs	Red blood cells
RDW	Red cell distribution width
RDW-CV	Red cell distribution width—coefficient of variation
RDW-SD	Red cell distribution width—standard deviation
PCV	Packed cell volume
PCT	Plateletcrit
PDW	Platelet distribution width
PLT	Platelet count
P-LCC	Platelet large cell count
P-LCR	Platelet large cell ratio
WBC	White blood cell count
WBCs	White blood cells
